# Understanding The Role of Lacticaseibacillus rhamnosus in Vaginal Dysbiosis: in Vitro Studies and Clinical Evidence

**DOI:** 10.1007/s12602-026-10922-1

**Published:** 2026-02-04

**Authors:** Benedetta Canala, Luciana Rossi

**Affiliations:** Department of Veterinary Medicine and Animal Sciences – DIVAS, University of Milan, Via Dell’Università 6, Lodi, 26900 Italy

**Keywords:** Vaginal dysbiosis, Microbiota, *L. rhamnosus*, Biosurfactants, In vitro study, Clinical trials

## Abstract

This narrative review aims to analyze and describe the outcomes of the most recent in vivo and in vitro studies involving *L. rhanmosus* in vaginal dysbiosis*.* Vaginal dysbiosis affects many women around the world and antibiotic treatment could not be a long-term solution due to the emergence of multidrug resistant microrganism. Considering that, probiotic treatments could represent an innovative and integrative approach to restore healthy vaginal microbiota. The dominant microbial population associated with vaginal health is represented by *Lactobacilli* spp.. Among all the *Lactobacillus* species, an interesting role is played by *L. rhamnosus,* able to produce several metabolites with antimicrobial properties such as lactic acid, bacteriocines, hydrogen peroxide and a class of molecules named biosurfactants. The in vitro studies on the main pathogens of vaginal dysbiosis (*G. vaginalis* spp., *Candida* spp. and HPV, HIV) showed coherent scientific evidence on the inhibitory, immunomodulatory and gene expression alteration effects of *L. rhamnosus* strains. Moreover, the promising preliminary outcomes were confirmed by clinical trials on infected women suggesting *L. rhamnosus* could be an interesting treatment in defense, protection and restoring of healthy vaginal microbiota, supporting women health.

## Introduction

The microbiota, a microbial community that hosts different districts of the human body, coexists with its genomic constitution and products in a mutualistic relationship [1]. Through the consistent contribution of the Human Microbiome Project (HMP) and Integrative HMP (iHMP), strongly supported by the National Institution of Health (NIH), the scientific research not only identified several microbiota communities, for instance, gut, epidermis, oral mucosa and vagina, but also investigated the characteristics, the distribution and the metagenomics of microorganisms that coexist in those anatomical districts [2, 3]. However, the findings of the cited projects revealed interesting aspects. On the one hand, microbiota eubiosis is crucial for the health status of the individual [4] considering its defensive activity, on the other hand microbiota is considered a dynamic community that could be disrupted facilitating pathogenic infections [5]. Vaginal microbiota is particularly affected by alterations: physiological (puberty, menstrual cycle, pregnancy and menopause) but also pathological (bacterial vaginosis, urinary tract infections and sexually transmitted infections) [6–8] or due to behavioural factors (smoking, antibiotics misuse, nutrition, sexual practices) [9] resulting in a dysbiotic profile. Nowadays, vaginal dysbiosis affects many women around the world and antibiotic treatment could not be a long-term solution considering the multidrug resistant microorganisms [10]. For this reason, as suggested by the World Health Organization (WHO), a reasonable and more sustainable alternative could be represented by the restoration of the vaginal beneficial microbial population [11]. The dominant microbial population associated with vaginal health is represented by *Lactobacilli* spp. [12]. Several studies highlighted the protective effects of *Lactobacilli* spp. through bacteriocins’ production, lactic acid and by competing with potentially pathogenic microorganisms blocking their biofilm formation using biosurfactants [13, 14]. These findings have led to an increase in *Lactobacillus* strains research in order to promote probiotics treatment as an innovative and integrative approach to pathogenic infections. Among all the *Lactobacillus* species isolated and selected for probiotics administration, an interesting role is played by *Lacticaseibacillus rhamnosus (L. rhamnosus)*. It is one of the earliest and most extensively studied probiotics within the *Lactobacillus* genus [15], moreover, it has well established potentialities in the treatment of several disorders such as, gastro-intestinal, paediatric, neurological, psychological and immunological [16–20]. However, the effect of *L. rhamnosus* on the improvement of vaginal dysbiosis was only recently investigated. Considering the complexity of the vaginal microbiological ecosystem and the disruptive role of bacterial, fungal and viral infections and the promising outcomes of the use of *L. rhamnosus* in other disorders, the strain could represent an innovative approach. Therefore, the following narrative review aims to explore *L. rhamnosus characteristics* emerged from in vitro studies and to analyse the impact of the strain administration in women affected by vaginal dysbiosis.

## Methods

All relevant published articles for the following narrative review were identified on PubMed and Scopus considering the period from 2009 up to 2024. The last literature search was updated to 2025. The literature search used the following key terms and their combinations: *L. rhamnosus*, vaginal dysbiosis, bacterial vaginosis, vulvovaginal candidiasis, sexually transmitted infections, in vitro studies, clinical trial. The keywords were combined with Boolean operators AND, OR in order to identify relevant studies on the investigation of *L. rhamnosus* in vaginal health. Among the considered papers in line with the selected time period, only peer-reviewed original studies evaluating the in vitro and in vivo treatment of vaginal dysbiosis with administration of *L. rhamnosus* strains, alone or in combination, were included. Manuscripts published outside temporal range and not related to vaginal dysbiosis and *L. rhamnosus* treatments were excluded. Considering the inclusion criteria, a total of 39 manuscripts were identified and 26 studies were included. The selected papers were critically discussed and summarized narratively to underline findings, limitations and future perspectives in the research.

## The Disruptive Role of *L. rhamnosus*’s Biosurfactants During Pathogens’ Biofilm Production

*L*. *rhamnosus* is the most studied lactic acid bacterium that was well-characterized for its probiotic effects. In general, the potential mechanisms considered responsible for the probiotic effects are mainly based on the competition for colonisation, production of lactic acid, release of organic acids and antimicrobial compounds and formation of a class of molecules with tensioactive and emulsifying properties called biosurfactants [21]. Over the past decades, biosurfactants have attracted the scientific research attention for their interesting biological role especially against biofilm production of some pathogenic microorganisms. In nature, microorganisms can live free floating in their planktonic form or aggregated in a sessile form. Depending on the environmental conditions, micro-organisms can switch from one to the other form, in a multi-step process that lead to the constitution of a defensive shield for the growing colony called biofilm [22]. Biofilm is a community of microorganisms that lives, grows and interacts in a matrix of extracellular polymeric substance (EPS) by sharing signalling and gene expression variations [23, 24]. Considering that, the biofilm is to be regarded as a dynamic and plastic environment that allows the survival and development of the microbial colony. However, the ability to form biofilms is also inherent to pathogenic microorganisms, often leading to complex clinical manifestations. For this reason, one of the strategies is to attempt biofilm disruption. Lactic bacteria, as *L. rhamnosus* strains, are able to produce several metabolites with antimicrobial properties such as lactic acid, bacteriocines, hydrogen peroxide and a class of molecules named biosurfactants. The biosurfactants inhibit the potential pathogens’ adhesion acting as potent antibiofilm agents [25]. At the very beginning of biosurfactants investigation, a study conducted of Howard et al. identified collagen-binding proteins in biosurfactants isolated by *Lactobacillus fermentum* RC-14, *L. casei* Shirota and *L. rhamnosus* GR-1 and 36 confirming an interaction of these probiotics with collagen of type III and type VI, structural proteins of animal tissues [26]. Starting from this outcome, more recent in vitro studies have attested the potential anti-adhesive and antibiofilm properties of *L. rhamnosus* strains biosurfactants against pathogens [27]. Isolated biosurfactants of *L. rhamnosus* ATCC7469 have impaired the attachment process and biofilm production of *S. mutans* strains. More in detail, the adhesion of *S. mutans* was reduced of 40% comparing with the pathogen growth control [28]. These outcomes opened the discussion on the possible mechanism of antibiofilm and antiadhesive processes especially considering that the incubation of S*. mutans* strains with biosurfactants had led to gene expression alteration. Interestingly, a reduction in gtfB, gtfC and ftf expression was recorded. These genes code respectively for the production of insoluble glucans, indispensable for tissue adhesion, and enzymes that could catalyse sucrose in order to obtain energy for the growing colony [29]. In addition, gtfB and gtfC are important virulence factor for the bacteria suggesting the interesting preventing role of biosurfactants against *S. mutans* biofilm production and development. Another significative example of antibiofilm and antiadhesive activities is represented by *L. rhamnosus* strains isolated biosurfactants described in the study of Sambanthamoorthy’s group. The biosurfactants were tested in order to inhibit host adhesion of clinically relevant multi-drug resistant pathogens such as *E. coli*, *S. aureus* and *A. baumannii*. It was found that the incubation of *L. rhamnosus strains’* biosurfactants at 50 mg/mL impaired the attachment of *E. coli* and *A. baumannii* and 25 mg/mL for *S. aureus* when compared to normal pathogen adhesion. In addition, the same dilutions seamed to be effective in dispersing biofilm formation especially when the incubation of *E. coli*, *S. aureus* and *A. baumannii* with the biosurfactants was prolongued. Besides the antiadhesive and antibiofilm properties, in the same study the *L. rhamnosus* biosurfactants have shown antimicrobial activity ranged from 96–97% against *A. baumannii*, 72–85% against *E. coli* and 80 and 93% for *S. aureus* strains UAMS-1 and MRSA respectively. Observations on transmission electron microscopy have also highlighted that most of the cells treated with biosurfactants were devoided of cell wall, the so called “ghost cell” phenomenon, suggesting that cell wall structure was strongly impaired leading to a leakage of cellular content. Moreover, several treated cells exhibited septa demonstrating biosurfactants could interfere with cell division process [30]. Antimicrobial activity of biosurfactants was also confirmed by an in vitro study on *L. rhamnosus* strains isolated from Iraqi healthy women vaginal environment. The results showed that the incubation of biosurfactants against the principal urinary tract infection pathogens that affect this female population such as *E. coli*, *K. pneumonia*, *B. cepacia* and *S. aureus*, caused the microbial inhibition at 64 mg/mL for *E. coli* and 32 mg/mL for the others [31]. Moreover, *L. rhamnosus* biosurfactants effect seems to exert antifungal properties [32]. dos Santos et al. reported the evaluation of the inhibitory effect of biosurfactants of a pool of *Lactobacillus* strains isolated from asymptomatic women, women affected by *Candida* spp. infection and reference strains on different *Candida* strains isolated from symptomatic and asymptomatic women. The results revealed that among the promising biosurfactants analysed, whom of *L. rhamnosus* ATCC 9595, a reference strain, were able to reduce *Candida* spp. strains adhesion of 78% and the biofilm formation of 30% [33]. Considering all the outcomes, is quite evident the presence of a correlation between antibiofilm – antiadhesive, antimicrobial activity and biosurfactants. However, a comprehensive understanding of biosurfactants’ mechanisms remains unclear. Scientific literature suggests that at the base there is emulsifying activity that reduce surface tension due to the fact biosurfactants are constituted by several compounds such as protein, polysaccharide, and phosphate; more specifically they are divided into low molecular mass compounds (glycolipids, lipopeptides, proteins) and high molecular mass compounds such as polysaccharides, lipoproteins, and polymeric particles type [34, 35]. For instance, lipopeptide could interfere with membrane composition and permeability altering its functions, the dimerization of surfactin in the phospholipidic bilayer could lead to integrity impairment or an unbalance in lipopolysaccharide content, caused by high content of rhamnolipids, could disrupt the surface morphology. Additionally, biosurfactants could interfere with quorum sensing signaling influencing the process of biofilm formation and cause apoptosis in several microbial cells by metabolic disruption [36]. Interestingly, a study on *L. rhamnosus* strains, isolated from human breast milk, demonstrated that the biosurfactants of these strains can reduce surface tension due to the formation of amphipathic aggregates called micelles; moreover, it was found that the biosurfactants were particularly effective against *B. subtilis*, *E. coli*, *P. aeruginosa* and *S. aureus* growth exhibiting a bactericidal potential at the following MIC and MBC dilutions: 12.5 and 25 mg/mL for *B. subtilis* and *E. coli*, 25 and 50 mg/mL for *P. aeruginosa* while 50 and 1000 mg/mL for *S. aureus*. At the same MIC values, biosurfactants of *L. rhamnosus* displayed also antibiofilm activity against the cited pathogens. Furthermore, the authors deeply investigated the effects of biosurfactants using SEM microscopy. The outcomes demonstrated the disruption of cell walls, the biofilm thickness strongly reduced and an altered pathogens’ morphology. In addition, the extracellular polymeric substances, essential for the biofilm instauration, were drastically impaired. From a molecular point of view, Gas Chromatography and Mass Spectrometry analysis collected a bunch of molecules responsible of antibiofilm and antimicrobial activities. Among all, Pyrrolo[1,2-a]pyrazine-1,4-dione,hexahydro-3-(phenylmethyl)-Cyclo(D-phenylalanyl-L-prolyl) is a potent growth inhibitor on S. mutans, the mannofuranoside and derivates exert strong antimicrobial activity against Gram negative and Gram-positive bacteria, 1-Heneicosanol, 9-Octadecene, (E)-, 1-Heptadecene, *n*-Hexadecanoic acid that affect different pathogenic bacteria and a yeast (*C. albicans*) [37]. Several studies have described biosurfactants’ effect of *L. rhamnosus* strains confirming their potential role in the treatment of pathogenic infections acting against biofilm formation, adhesion processes and proliferation. Furthermore, scientific literature attested the molecular potential of biosurfactants in order to consider them as innovative parameters in lactobacilli probiotics selection.

## *L. rhamnosus* in Vaginal Dysbiosis: in Vitro Studies

Vaginal dysbiosis represents a microbial community impairment usually affecting the lactobacilli population. This fluctuation could lead to an increase in anaerobic microorganism and fungi even exposing women to severe infections [38]. Among all, bacterial vaginosis (BV), vulvovaginal candidiasis (VVC) and sexually transmitted infections (STI) are the main identified female genital tract disorders. Nowadays, the antibiotic treatment or, when identified a fungal infection, antifungal administration, is considered the first defence against infections. Nevertheless, this approach is often related to high incidence of recurrency and enhancing of the risk of antimicrobial resistance rising [39]. Considering this concern, probiotics supplements are meant to be an alternative approach [40], but the identification and approval of putative strains need a consistent background of scientific evidence [41]. *L. rhamnosus* strains have been deeply in vitro investigated for their probiotic activity and safety profile [42–46]. However, it is interesting to discuss the main in vitro outcomes on *L. rhamnosus* strains involvement against the main infective pathogens (Table 1). Samples of vaginal swabs recovered from women affected by BV are usually positive for high presence of *Gardnerella vaginalis*. From literature, several in vitro studies have described the effects of incubating strains of *L. rhamnosus* with *G. vaginalis*. More specifically, in a study on *L. rhamnosus* GG, it was found that the strain was not only able to inhibit biofilm formation of *G. vaginalis* but was also able to disaggregate preformed biofilms and reduce the bacterial load by exerting antimicrobial activity [47]. In particular, the antimicrobial property of the strain, exhibited by bacteriocins’ production, seemed to be more expressed against the preformed biofilm of *G. vaginalis* suggesting a possible ability to interfere with the pathogen's biofilm production process. However, the results obtained in the in vitro study by He et al. [20] showed that incubation of *L. rhamnosus* Xbb-LR-1 with *G. vaginalis* appeared to have the strongest inhibitory capacity at the beginning of pathogen biofilm formation [48]. The antimicrobial activity against *G. vaginalis* was also evaluated in the strain *L. rhamnosus* RD-0060 isolated from vaginal secretions of healthy women suggesting that defensive molecules such as acetic acid and lactic acid, produced by strain, compromised the integrity of the cell membrane, affected the normal membrane potential and ATP synthesis of the microorganism, reduced the activity of Na +/K + -ATPase, and inhibited the metabolism and the normal growth of *G. vaginalis* [49]. In addition, *L. rhamnosus* strains’ incubation with *G. vaginalis* appears to exert immunomodulatory functions. In an in vitro study, Miquel et al. [41] evaluated the incubation of *Lacticaseibacillus rhamnosus* Lcr35 (Lcr35) with the vaginal epithelial cell line (VK2/E6E7) and dendritic cell lines (DC). The DC line is responsible for the adaptive immune response; in order to mimic the vaginal epithelial barrier, the dendritic cells were cultured in an adjacent chamber. The results showed that, during *G. vaginalis* infections, Lcr35 was able to indirectly stimulate the activity of DCs that released cytokines and chemokines such as CCL20 while the VK2/E6E7 line increased the production of HBD-2 (antimicrobial peptide), IL-8 and IL-1β, well known to locally recall neutrophils. Nevertheless, when infection with *G. vaginalis* alone was considered, VK2/E6E7 cell line produced secretory leukocyte protease inhibitor (SLPI), molecules with antimicrobial activity. SLPI production appeared to be inhibited with the incubation of Lcr35 suggesting a preference of the strain in activating NF-kB pathway that induces immunostimulation through a basal inflammatory state typical of vaginal environments with a preventive protective function [50]. The importance of immune modulation by lactobacilli helps in the prevention and inhibition of possible migratory pathogens such as, for example, *E. coli* UPEC strains that cause urogenital disorders and damage [51] but also in the containment of other microorganisms that under microbial community eubiosis establish symbiotic relationships with the environment. Like in the case of *Candida* spp*.*, commensal fungi of vaginal mycoflora [52]. When *Candida* spp*.*, evolve from colonization to symptomatic infection due to fungal spread, a vulvovaginal candidiasis (VVC) is established. In a in vitro study*, L. rhamnosus* GG incubated with *C. albicans*, the most frequent species implicated in VVC, interfered with fungal growth; in particular, the isolated EPS from *L. rhamnosus* GG reduced hyphal formation by almost 50%. Purified EPS itself inhibited adhesion, supporting its role in competing with Candida for epithelial binding [53]. In addition, Kohler et al. [30] connected the strong inhibitory activity of L. rhamnosus GR-1 to antimicrobial substances and lactic acid production. In fact, in the cited study, the incubation of C. albicans with the probiotic surnatants showed high inhibitory rate while the incubation with filtered surnatants abrogated the effect [54]. Moreover, the discussed results were obtained even in the study of Inturri et al. [24] where the antimicrobial effects of *L. rhamnosus* HN001, alone and co-incubated with Bifidobacterium longum BB536, were attributed to metabolites such as lactic acid, acetic acid, small peptides, and bacteriocins released by the probiotics. These metabolites, particularly at low pH, showed strong activity against bacteria and *Candida* spp. [55]. However, the inhibition of *L. rhamnosus* occurs through more complex mechanisms. Results on in vitro models of intestinal cells suggest that the protective effect seems to be induced not by supernatants but by *Lactobacillus*-fungus contact, which inhibits *Candida* hyphal formation, together with apoptotic infected cells, and translocation, i.e. the passage of the fungus within the cell [56]. Furthermore, colonisation by *L. rhamnosu*s strains appears to alter the availability of nutrients in the vaginal environment. In fact, probiotic strains consume available molecules such as glucose and amino acids, forcing the fungus to feed on other compounds, mainly lactate and malate. As a result, *C. albicans* changes basal metabolism but, consequently, reduces virulence. It is caused by altered gene expression, with down-regulation of genes associated with virulence and invasiveness of the fungus [57]. These results are in line with the previous study by Rossoni et al. [59] who demonstrated that the inhibitory effects of Co-incubation between *L. paracasei* 28.4, *L. rhamnosus* 5.2 and *L. fermentum* 20.4 against *C. albicans* influence expression of fungal biofilm-specific genes (ALS3, HWP1, EFG1 and CPH1) resulting in a deterrence of biofilm development and retardation of hyphal formation [58]. Besides the possible modulation effects of *C. albicans* gene expression, it appears that incubation with lactobacilli may modulate the immune response. More in detail, the combination of *L. reuteri* RC-14 and *L. rhamnosus* GR-1 increased IL-8 and IP-10 production in VK2/E6E7 epithelial vaginal cells infected with *C. albicans*, suggesting that probiotics may enhance immune responses during infection. Moreover, direct contact with probiotics also up-regulated the gene expression of IL-8 and IP-10 [59]. The relevant protective and immune modulatory effects of *L. rhamnosus* spp. are both established in BV and VVC suggesting un possible and promising key role also in the prevention and reduction of sexually transmitted infections (STI). The in vitro studies focused on the interaction of *L. rhamnosus* spp. with the main sexually transmitted virus (HPV and HIV) are few but consistent in the outcomes. Motevaseli et al. [42] clarified the mechanism by which lactobacilli exert cytotoxic effect and onco-genes regulation during HPV infection in HeLa cell line model. The infection of human papilloma virus (HPV) is the main cause of cervical cancer onset in women. At cellular level, in order to defend and eliminate the abnormal cells, autophagy process takes place. In the cited study, the HPV18 DNA infected HeLa cell line is able to express HPV E6-E7 onco-genes; the incubation of cell line with the surnatant of *L. rhamnosus* GG culture exhibited inhibitory effect against infected HeLa cells compared to culture medium and culture medium enriched in lactate suggesting that the anti-proliferative effect did not depend only on the lactic acid production. Moreover, *L. rhamnosus* GG surnatant helped to downregulate HPV-E6 oncogene demonstrating that the favorable mechanism of inhibition passes through a gene expression modulation [60]. The antiproliferative effect, but not the virucidal effect, was even observed against HIV in vitro. The incubation of *L. rhamnosus* spp. surnatants with HIV virion replicons demonstrated high inhibitory effects on viral replication achieving more than 50% of viral multiplication reduction [61]. However, an interesting aspect in the evaluation of *L.rhamnosus* spp. is represented by its involvement in prevention plans. A vaccination plan against HIV does not exist properly; nowadays, the therapeutic approach is based on passive immunization through broadly neutralizing antibodies or by using nanobodies (VHH), smaller and more stable antibodies derived from camelids. Unfortunately, VHH exhibit a short half-life, thus they need a vehicle in order to stay longer in the vaginal mucosa. In the in vitro study of Kalusche et al. [28] *L. rhamnosus* DSM 14870 was modified in order to deliver VHHA6 in the vagina. The outcomes showed that around 40% *L. rhamnosus* DSM 14870 population expressed nanobodies on the surface and that VHHA6 neutralized several HIV-1 sierotypes. It was confirmed by fluorescently labelled HIV-1 bound to lactobacilli expressing VHHA6 [62]. The in vitro studies on the main pathogens of vaginal dysbiosis showed coherent scientific evidence on the inhibitory, immunomodulatory and gene expression alteration effects of *L. rhamnosus* strains highlighting the promising probiotic role of defense and protection of healthy vaginal microbiota.

**Table 1:** In vitro selected studies on *L. rhamnosus* spp. properties from 2009 to 2023.

Article	Strain	Primary Outcomes	Reference
Anti-Biofilm Properties of Saccharomyces cerevisiae CNCM I-3856 and *Lacticaseibacillus rhamnosus* ATCC 53103 Probiotics against G. vaginalis	*L. rhamnosus GG*, *S. cerevisiae* CNCM I-3856 +	*L. rhamnosus GG* and *S. cerevisiae* CNCM I-3856 + can inhibit biofilm formation, reduce bacterial viability within the biofilm and promote biofilm disaggregation	Sabbatini et al. [63]^47^
*Lactobacillus rhamnosus* *and Lactobacillus casei* Affect Various Stages of Gardnerella Species Biofilm Formation	*L. rhamnosus, L. casei*	*Gardenerella* biofilm was impaired by lactic acid lactobacilli strains production	He et al. [20]^48^
Novel Potential Probiotic Lactobacilli for Prevention and Treatment of Vulvovaginal Infections	*L. rhamnosus* RD-0060	*L. rhamnosus production of* acetic and lactic acids disrupted bacterial cell membrane integrity, altered membrane potential and ATP synthesis, reduced Na⁺/K⁺-ATPase activity, and ultimately inhibited the metabolism, growth, and reproduction of *G. vaginalis*	Huang et al. [22]^49^
*Lacticaseibacillus rhamnosus* Lcr35 Stimulates Epithelial Vaginal Defenses upon Gardnerella vaginalis Infection	*L. rhamnosus* Lcr35	*Lcr35* exhibited an immunostimulatory effect by activating both epithelial and dendritic cell responses	Miquel et al. [41]^50^
The lectin-like protein 1 in *Lactobacillus rhamnosus* GR-1 mediates tissue-specific adherence to vaginal epithelium and inhibits urogenital pathogens	*L. rhamnosus* GR-1	The lectin domains of *L. rhamnosus GR-1* strongly affected the adhesion and biofilm formation of *E. coli* UTI89	Petrova et al. [50]^51^
Interplay between *Lactobacillus rhamnosus* GG and Candida and the involvement of exopolysaccharides	*L.rhamnosus* GG	*Lactobacillus rhamnosus GG* can interfere with *Candida albicans* growth. Its exopolysaccharides (EPS) prolong the lag phase of *Candida* growth but do not strongly inhibit overall growth. *L. rhamnosus GG* EPS significantly reduces hyphal formation (~ 50%) and inhibits adhesion, indicating a role in competing with *Candida* for epithelial binding	Allonsius et al. [1]^53^
Probiotic interference of *Lactobacillus rhamnosus* GR-1 and *Lactobacillus reuteri* RC-14 with the opportunistic fungal pathogen Candida albicans	*L. rhamnosus* GR-1, *L. reuteri* RC-14	*L. rhamnosus GR-1* and *L. reuteri RC-14* inhibit *Candida albicans* growth at low pH. L. rhamnosus GR-1 suppressed biofilm formation of C. albicans	Kohler et al. [30]^54^
In vitro inhibitory activity of *Bifidobacterium longum* BB536 and *Lactobacillus rhamnosus* HN001 alone or in combination against bacterial and Candida reference strains and clinical isolates	*Bifidobacterium longum* BB536, *Lactobacillus rhamnosus* HN001	The supernatants from broth cultures of *B. longum* and *L. rhamnosus*, showed antimicrobial activity. These supernatants also reduced adhesion of clinical bacterial isolates to HT-29 human intestinal cells, suggesting potential for infection prevention. The antimicrobial effects were attributed to metabolites such as lactic acid, acetic acid, small peptides, and bacteriocins	Inturri et al. [24]^55^
Keeping *Candida* commensal: how lactobacilli antagonize pathogenicity of *Candida albicans* in an in vitro gut model	*L. paracasei, L. rhamnosus*, *L. salivarius*,* L. fermentum/L. brevis*	*L. rhamnosus* reduced C. albicans translocation and hyphal elongation	Graf et al. [29]^56^
*Lactobacillus rhamnosus* colonisation antagonizes Candida albicans by forcing metabolic adaptations that compromise pathogenicity	*L. rhamnosus* spp*.*	*L. rhamnosus* produces metabolites that further affect the growth and survival of C. albicans	Alonso-Roman et al. [2]^57^
Antifungal activity of clinical *Lactobacillus* strains against *Candida* albicans biofilms: identification of potential probiotic candidates to prevent oral candidiasis	*L paracasei 28.4*, *L. rhamnosus 5.2*, *L. fermentum 20.4*	*L. paracasei 28.4, L. rhamnosus 5.2, and L. fermentum 20.4* showed the strongest inhibitory activity against *C. albicans*, inhibited biofilm formation and slowed hyphal development	Rossoni et al. [59]^58^
Effect of *Lactobacillus rhamnosus* GR-1 and *Lactobacillus reuteri* RC-14 on the ability of *Candida albicans* to infect cells and induce inflammation	*Lactobacillus rhamnosus* GR-1, *Lactobacillus reuteri* RC-14	*L. reuteri RC-14 and L. rhamnosus GR-1 cell-free supernatants inhibited C. albicans* growth at 24 h. Biofilm inhibition is likely due to nutrient competition and production of anti-Candida compounds, with L. rhamnosus GR-1 specifically suppressing biofilm formation, a key factor in *Candida* virulence	Martinez et al. [40]^59^
*Lactobacilli* Expressing Broadly Neutralizing Nanobodies against HIV-1 as Potential Vectors for HIV-1 Prophylaxis?	*L. rhamnosus* DSM 14870	*Lactobacillus rhamnosus DSM 14870* expressed VHHA6 nanobodies in order to bind HIV-1 particles and prevent the infection	Kalusche et al. [28]^62^

## *L. rhamnosus* and Vaginal Dysbiosis: Clinical Evidence

Clinical evidence is at the base of probiotics’ validation and assessment. Starting from the promising outcomes obtained by in vitro studies, in vivo investigation aims to clarify the survival and the effects of *L. rhamnosus* activity at systemic level [63, 64] (Table 2). On the one hand, clinical trials give information on the effective presence of the administered *Lactobacillus* strain at vaginal level; on the other hand, allow to evaluate the efficacy on the principal vaginal dysbiosis. In the clinical trial of De Alberti et al. [13], during the oral administration of *L. rhamnosus* HN001, *L. acidophilus* La-14 and bovine lactoferrin for 14 days, vaginal swabs were collected every week up to 21 days in order to identify the supplemented strains through gene sequencing. The procedure was conducted among the treatment and placebo group and the outcomes confirmed a higher increase of *L. rhamnosus* HN001 and *L. acidophilus* La-14 population in the treated group from the second week of administration up to one week after the end of the assumption. More in detail, *L. rhamnosus* HN001 exhibited a significative growth in the treatment group when compared to the starting point of the trial [65]. The described results demonstrated that *L. rhamnosus* HN001 was detected in the samples suggesting that is able to survive in the vaginal environment. Considering that, *L. rhamnosus* HN001 colonize and develop increasing its growth rate. Nevertheless, the most important aspect of in vivo trials is the possibility to explore the direct effects on human health. Nowadays, probiotic administration could represent therapeutic option mainly in case of vaginal dysbiosis recurrency. In fact, despite the treatments, women could experience recurring episodes of infection up to 58% over the course of 12 months [66]. In addition, recurrency negatively affects physical, psychological and relational well-being of women [67, 68]. From literature, the use of *L. rhamnosus* strains has been already investigated with positive outcomes on BV management, restoring the physiological vaginal pH and attenuating related symptoms [69]. More recently, in order to enforce preliminary results on the use of lactobacilli in vaginal dysbiosis, several studies have demonstrated the effects of the supplementation of strains besides to antibiotic treatment. In a study of Marcone et al. [37] women population affected by BV was randomly divided into two experimental groups: group A treated with twice daily oral administration of metronidazole (500 mg) for 7 days, group B followed the same antibiotic therapy followed, after 8 days of the end of the treatment, by once daily vaginal application of *L. rhamnosus* capsule (40 mg) for 6 months. The study aimed to demonstrate the impact of the use of lactobacilli in BV treatment following antibiotic administration. Although the study had some limitations such as the low numbers (group A: 25 women and group B: 24 women) and the lack of data at baseline, follow-up at 30, 90, 180, 270 and 360 days showed around 20% higher rate of vaginosis free patients in group B than group A treated with antibiotics only. Notably, vaginosis episodes did not occur during the 6 months of probiotic treatment nor in the 6 months of follow-up suggesting that the use of *L. rhamnosus* ensured to restore and sustain the stability of the microbial ecosystem [70]. These results were also confirmed by the study of Recine et al. in 2015. Compared to the previous one, Recine et al. provided more precise indications by introducing the *Lactobacillus* strain used (*L. rhamnosus* BMX 54) and increased the statistical power with a higher numerosity (250 women with BV). The sampling and analyses performed after 2 months, 6 and 9 months were the same as in Marcone's group such as vaginal fluid collection, pH assessment, Whiff test and secretion and symptoms evaluation. The study reinforced the result on the stability of the microbiota treated with *L. rhamnosus* BMX 54 showing a symptomatic improvement in 92% of patients compared to 79% of patients treated with antibiotics only at 9 months of follow up. Moreover, the pH of group A rose up to 5.5 during the follow up suggesting a negative correlation between high vaginal pH and BV [71]. Nevertheless, the cited studies did not discuss the possible symptoms relief after the treatment. In fact, bacterial vaginosis causes itching, burning sensations and vaginal discharge affecting the quality life of women. In the double blind, randomized, placebo controlled clinical trial study of Russo et al. [62], women with abnormal microbiota at Nugent test exhibiting vaginal dysbiosis similar to BV, were randomly divided into treatment group and placebo group. In the treatment group, a blend of lactobacilli (5 × 109 CFU of *Lactobacillus acidophilus* GLA-14 and *Lactobacillus rhamnosus* HN001) and prebiotics (bovine lactoferrin 40 mg) were administered for 15 days. Interestingly, Nugent score, a useful tool to evaluate vaginal microbiota through Gram staining, had an improvement at the end of the administration in treated group suggesting a restoration of healthy microbiota. Moreover, the study provided data on the amelioration of vaginal symptoms: in particular, the treatment exhibited a relief in itching and vaginal discharge compared to placebo group. These outcomes were supported by the colonization of the strains in the blend, in fact, genetic analysis on vaginal samples have demonstrated the presence of *Lactobacillus acidophilus* GLA-14 and *Lactobacillus rhamnosus* HN001 in the microbiota composition of treated women [72]. The symptoms improvement due to lactobacilli supplementation could positively affect women quality life. As reported in literature, the oral administration for 10 days of *L. rhamnosus* CA15 in women with vaginal dysbiosis led to statistically significant reduction of self-reported symptoms during the trial lasted, in detail, an improvement of itching, burning, leucorrhea, vaginal discomfort and vaginal edema. The symptomatic improvements presented are confirmed by the quality life assessment of the treated patients after 30 days after the end of the treatment; more in detail, the questionnaire resulted significative in the perception of the environment, physical health and social relations of the women suggesting that the *L. rhamnosus* CA15 administration positively affect the quality life. In fact, even the question on the overall quality of life was significative in the treatment group [73]. The results of lactobacilli’ impact on improving quality of life were also discussed in the clinical trial by Vaccalluzzo et al. [76], in which *L. rhamnosus* TOM 22.8 strain isolated from healthy women, known for its adhesiveness, antioxidant and anti-inflammatory properties and colonisation [74], administered to a dysbiotic population for 10 days, led to an improvement in symptoms already after the aforementioned treatment period. Furthermore, 30 days after the end of treatment, all 60 women recruited in the treatment group reported a recovery of vaginal eubiosis compared to the control group [75]. The discussed outcomes underlined that *L. rhamnosus* administration may contribute to improve quality life in women affected by vaginal dysbiosis. However, there are some methodological limitations in the available clinical trial, such as sample size, lack of placebo control and follow-up periods. These elements must be considered in the interpretation of the obtained results and future well-designed, long-term and placebo-controlled studies should confirm the promising results. However, an interesting finding from this study concerns the reduction in the cell density of *Candida* spp. in the vaginal samples analysed. In fact, the *L. rhamnosus* TOM 22.8 strain was able to counteract the growth and development of several *Candida* species (e.g. *C. glabrata*, *C. albicans*, *and C. krusei*) both during administration and during follow-up. The interesting contribution against *Candida* spp. opens the possibility of using *L. rhamnosus* in the treatment of VVC. The main recommended therapy for candidiasis is antifungal drugs. However, improper and often self-administered use of the medication can lead to poor efficacy, especially in the long term, exposing patients to annoying recurrences. Considering this, the use of antifungal drugs remains a topic for further study. In fact, even considering populations of women with acute forms of VVC in which treatment is necessary, it has been assessed that, in the long term, antifungal drugs are capable of depleting the lactobacilli load in the vaginal microbiota, a prerequisite for a healthy vaginal microbiota [76]. In the clinical trial by Russo et al. [62], the blend of lactobacilli and lactoferrin discussed for BV was also tested in women with recurrent VVC. During the trial, antifungal drug (clotrimazole 100 mg daily for 7 days) was administered to patients and then they were randomly assigned to the treatment group (Lactobacilli blend and lactoferrin) or placebo group (2 capsules/day for 5 days than 10 consecutive days at the dosage of 1 capsule/day for 6 months followed the antifungal administration). Interestingly, treatment with probiotics and lactoferrin, when compared to placebo, reduced significantly candidiasis’s recurrency. More in detail, the results showed a significant reduction in the recurrency rate corresponding to 33.3% and 29.2% at 3 months and 6 months after the end of antifungal therapy [77]. Clinical studies conducted on patients with BV and VVC have shown that restoring the dominance of lactobacilli is positively linked to a recovery of the vaginal microbiota but, above all, to a reduction in recurrences. In fact, bacterial and fungal dysbiosis predispose women to viral infections, which are often sexually transmitted. Among these, several strains of HPV are associated with cancerous lesions of the cervix. There are different degrees of lesion, depending on which specific therapeutic approaches are undertaken and, in the most serious cases, even surgical ones. However, for low-grade lesions (CIN 1), surgery is not recommended in the first instance, considering that they may be transient infections. Considering that, several clinical trials have tested the possible benefits of administering lactobacilli. In particular, prolonged treatment with *L. rhamnosus* BMX54 appears to have been effective in resolving HPV-related cytological anomalies. Specifically, the study compared the different durations of oral probiotic treatment in a population affected by mild lesions, with one group receiving 3 months of probiotic strain and the other 6 months. The results of the follow-up, which lasted an average of 14 months, showed there was an improvement in lesions in both groups, approximately 11.6% in the short schedule probiotics group and 31.2% in the long-term vaginal Lactobacilli users [78]. Although studies confirm that the administration of lactobacilli is a valid therapeutic approach, especially in the long term, an interesting aspect that needs to be further investigated in vivo is the possible immunomodulatory effect, especially in cases where the immune system may be compromised by viral infections such as HIV. Few studies have been conducted on HIV-infected populations, but preliminary data on the administration of probiotic blends such as *L. rhamnosus* GR-1 and *L. reuteri* RC-14 [79] or *L. rhamnosus* HN001 and *B. lactis* Bi-07 [17] have shown a significant increase in CD4 + lymphocytes in treated patients, suggesting immune strengthening. Although the studies are few and not recent, the results could be a first step towards investigating an innovative and interesting aspect that probiotic research has opened to the scientific world [46, 64].

**Table 2:** In vivo selected studies on *L. rhamnosus* spp. supplementation from 2010 to 2024.

Article	Study Details	Primary Outcomes	Reference
*Lactobacilli* vaginal colonisation after oral consumption of Respecta complex: a randomised controlled pilot study	• *L. acidophilus* La-14, *L. rhamnosus* HN001(10 × 10^9^ CFU/die) and bovine lactoferrin (50 mg) • Sample size: 40 women • 2 weeks, oral capsules, twice daily	Consumption of *L. acidophilus La-14, L. rhamnosus HN001* in combination with bovine lactoferrin leads to vaginal detection; even 1 week after consumption was stopped	De Alberti et al. [13]^65^
The use of *Lactobacillus rhamnosus* in the therapy of bacterial vaginosis. Evaluation of clinical efficacy in a population of 40 women treated for 24 months	• *L. rhamnosus* BMX 54 (40 mg – CFU not reported) • Sample size: 40 women • 24 months of vaginal tablets administration	*L. rhamnosus* BMX 54 restored the physiological vaginal pH and controlled BV symptoms, and supports the use of pH measurement for sensitive, objective, and simple therapy follow-up in women with BV	Rossi et al. [58]^69^
Long-term vaginal administration of *Lactobacillus rhamnosus* as a complementary approach to management of bacterial vaginosis	• *L. rhamnosus* BMX 54 (40 mg—CFU not reported) • Sample size: 40 women • 6 months of vaginal tablets administration once a week	he vaginal administration of the probiotic *L. rhamnosus* BMX 54 allows stabilization of the vaginal ecosystem	Marcone et al. [37]^70^
Restoring vaginal microbiota: biological control of bacterial vaginosis. A prospective case–control study using *Lactobacillus rhamnosus* BMX 54 as adjuvant treatment against bacterial vaginosis	• *L. rhamnosus* BMX 54 (dosage not reported) • Sample size: 125 women control group (antibiotic treatment alone),125 women treated with antibiotic and vaginal tablet once a day • 9 months follow up (evaluation at 2–6–9 month)	Probiotic supplementation with vaginal *Lactobacillus rhamnosus* BMX 54 seems to be useful in hindering bacteria growth especially after antibiotic therapy therefore this intervention may be considered a new prophylactic	Recine et al. [57]^71^
Study on the effects of an oral *lactobacilli* and lactoferrin complex in women with intermediate vaginal microbiota	• *Lactobacillus acidophilus* GLA-14 (5 × 10^9^ CFU), *Lactobacillus rhamnosus* HN001(5 × 10^9^ CFU) and bovine lactoferrin (50 mg) • Sample size: 48 women divided into treatment and placebo groups • 15 days of oral capsules once a day	The mixture significantly improved Nugent scores and alleviated symptoms such as itching and vaginal discharge in women with symptomatic vaginal dysbiosis	Russo et al. [62]^72^
*Lacticaseibacillus rhamnosus* CA15 (DSM 33960) strain as a new driver in restoring the normal vaginal microbiota: A randomized, double-blind, placebo-controlled clinical trial	• *L. rhamnosus* CA15 (DSM 33960) (1 × 10^10^ CFU) • Sample size: 60 women • 10 days of oral capsules administration once a day	Improvement of quality of life in women with vaginal dysbiosis	Rapisarda et al. [55]^73^
A clinical pilot study on the efect of the probiotic *Lacticaseibacillus rhamnosus* TOM 22.8 strain in women with vaginal dysbiosis	• *L. rhamnosus* TOM 22.8 (1 × 10^1^⁰ CFU/g) • Sample size: 30 women divided into oral, vaginal administration and wait and see • 10 days once a day	The strain showed broad antagonistic activity against vaginal pathogens, strong adhesion to vaginal (VK2/E6E7) and intestinal (Caco-2) cells, and anti-inflammatory and antioxidant effects. Both oral and vaginal administration led to a significant reduction of pathogens after 10 days and maintained microbial eubiosis for up to 30 days after treatment ended	Pino et al. [52]^74^
*Lacticaseibacillus rhamnosus* TOM 22.8 (DSM 33500) is an effective strategy for managing vaginal dysbiosis, rising the lactobacilli population	• *L. rhamnosus* TOM 22.8 (10 × 10^9^ CFU) • Sample size: 80 women • 10 days of oral capsules administration once a day	Thestrain restored physiological vaginal pH, accompanied by remission or reduction of clinical signs and symptoms and improved quality of life (QoL). Microbiological analyses showed a significant reduction in potentially pathogenic bacteria	Vaccalluzzo et al. [76]^75^
Impact of a *lactobacilli*-containing gel on vulvovaginal candidosis and the vaginal microbiome	• *L. rhamnosus* GG, *L. pentosus* KCA1 and *L. plantarum* WCFS1 (10⁹–10^1^⁰ CFU/g gel) • Sample size: 20 women • 10 day of intravaginal gel application once a day	The probiotic gel was effective in milder cases of vaginal infection, providing rapid symptom relief even without additional treatments	Oerlemans et al. [47]^76^
Randomised clinical trial in women with Recurrent Vulvovaginal Candidiasis: Efficacy of probiotics and lactoferrin as maintenance treatment	• *Lactobacillus acidophilus* GLA-14, *Lactobacillus rhamnosus *HN001 (5 × 10^9^ CFU/capsule of probiotc mix), bovine lactoferrin (50 mg) • Sample size: 48 women • 6 months of oral capsules administration (Induction phase: 2 capsules/day for 5 days than 1 capsule/day for 10 days. Maintenance phase: 1 capsule/day for 10 days a month for 6 months)	After clotrimazole therapy, both groups improved, but only women receiving probiotics with lactoferrin showed sustained relief from itching and discharge at 3 and 6 months. Recurrences were significantly lower in treatment group compared to placebo	Russo et al. [61]^77^
Long-term *Lactobacillus rhamnosus* BMX 54 application to restore a balanced vaginal ecosystem: a promising solution against HPV-infection	• *L. rhamnosus* BMX 54 (1 × 10^4^ CFU/tablet) • *Sample size. 117 women divided into short term treatment and long term treament* • Short term: 1 tablet once a day for 10 days followed by 1 tablet every 3 days for 20 days and 1 tablet every 5 days for 2 months. Long term: 1 tablet a week for 6 months	*Lactobacilli* could help resolve cervical abnormalities by restoring physiological vaginal balance (eubiosis)	Palma et al. [48]^78^
Effect of 25 weeks probiotic supplementation on immune function of HIV patients	• *L. rhamnosus* GR-1, *L. reuteri* RC-14 (2 × 10^9^ CFU) • Sample size: 65 women divided into control and treatment groups • 25 weeks of oral capsule administration twice a day	Improved CD4 + count	Hummelen et al. [23]^79^
Synbiotic therapy decreases microbial translocation and inflammation and improves immunological status in HIV-infected patients: a double-blind randomized controlled pilot trial	• *L.rhamnosus* HN001, *B. lactis* Bi-07 (10^9^ CFU/mL) • Sample size: 20 women • 16 weeks of drop oral administration once a day	Increased beneficial gut bacteria and reduced harmful ones. CD4 + count was increased and IL-6 levels decreased	Gonzalez-Hernandez et al. [17]^80^

## Conclusions

Traditional treatments for vaginal dysbiosis are mainly antibiotics and antifungals. However, although they have a beneficial effect, in the long term they can neglect the need to restore the vaginal microbiota, exposing patients to recurrent episodes. For this reason, the administration of lactobacilli could represent an innovative and complementary approach. *L. rhamnosus* is among the most studied lactobacilli, and its ability to produce biosurfactants capable of inhibiting the formation of pathogenic biofilms makes it a valid candidate in the treatment of vaginal infections. In fact, evidence from recent in vitro and in vivo studies has shown that *L. rhamnosus* strains exert interesting antimicrobial, immunomodulatory and gene expression regulatory properties on virulence-associated factors, and have been related to improvement in patient symptoms suggesting a potential role in reducing the recurrence rate. Nevertheless, current evidence is mainly derived from studies in which probiotic combinations are involved. Therefore, further in vivo investigations on the efficacy of *L. rhamnosus* single strain are needed to clarify its contribution and therapeutic potential in vaginal dysbiosis.

## Data Availability

No datasets were generated or analysed during the current study.
